# Supplementary material for: Automated relative binding free energy calculations from SMILES to ΔΔG
Source: Commun Chem. 2023 Apr 27;6:82. doi: 10.1038/s42004-023-00859-9 (PMC10140266; doi:10.1038/s42004-023-00859-9)
Supplement: Supplementary file 2 — Supplementary Information [file 42004_2023_859_MOESM2_ESM.pdf]

Supplementary Information: Automated relative binding free energy calculations from SMILES to $\Delta\Delta$G

J. Harry Moore[1], Christian Margreitter[1], Jon Paul Janet[1], Ola Engkvist[1,*], Bert L. de Groot[2,*], and Vytautas Gapsys[2,*]

[1]Molecular AI, Discovery Sciences, R&D, AstraZeneca, Gothenburg, Sweden
[2]Computational Biomolecular Dynamics Group, Department of Theoretical and Computational Biophysics, Max Planck Institute for Multidisciplinary Sciences, Am Fassberg 11, D-37077, Göttingen, Germany
[*] Ola.Engkvist@astrazeneca.com, bgroot@gwdg.de, vgapsys@gwdg.de

Contents

1 Introduction **2**

1.1 Getting Started 2

1.1.1 Source Code 2

1.1.2 Conda Environment 2

1.1.3 Config File 2

1.1.4 Compound handling 5

2 Workflow Recipes **7**

2.1 Docking 7

2.2 Automated SMILES-to-ΔΔG 7

3 Workflow Steps **11**

3.1 Input / Output 11

3.1.1 Data Manipulation 11

3.2 Molecular Docking 11

3.2.1 RDKit Embedding 11

3.2.2 LigPrep 12

3.2.3 AutoDock Vina 13

3.2.4 Glide 16

3.3 Free Energy Methods 18

3.3.1 Perturbation Map Generation 18

3.3.2 PMX 19

4 Implementation Details **26**

4.1 Data Handling 26

4.1.1 Compound Objects 26

4.1.2 GenericData 26

4.2 Execution 26

4.3 Parallelization 27

Supplementary Note 1

Introduction

Icolos is a workflow manager for computational chemistry and cheminformatics. It is designed for rapid design and efficient execution of complex workflows. Most common workflows can be run 'out-of-the-box' from the provided templates, and can be further modified to suit individual use cases. Workflows are specified entirely using `JSON` files, and are built from a set of steps, through which data flows during execution.

The Icolos philosophy is built on a separation of workflow definition and implementation, whilst retaining complete and direct control over the step's execution. As a result, the user should never have to consider the implementation details further than which switches and options to set in the configuration.

1.1 Getting Started

The following section is designed to get new users up and running with some straightforwrd workflows, and introduce the major features of Icolos. Only the surface level complexity is discussed, with more detailed treatment of each component provided in **Section 4**.

1.1.1 Source Code

Icolos is entirely written in Python 3 and can be installed from PyPI or GitHub. Installation of the package using pip provides the `icolos` entrypoint to run a workflow i.e.

```
icolos -conf /path/to/config.json
```

1.1.2 Conda Environment

Icolos is written entirely in Python and its dependencies are packaged into an environment using conda. An environment specification file is provided in the repo from which the env can be built.

1.1.3 Config File

The configuration file consists of two major components:

- A Header (workflow-wide specifications such as environment variables) and
- The workflow logic, comprised of steps

Header

An example header is reproduced below. Unique workflow ID and description are set (only for book keeping) and define environment variables to be exported. Global variables and settings (that affect the execution of the entire workflow) are also set in the header. Note that Icolos automatically sets the following global variables relating to the runtime settings, and these can be used to specify filepaths without further modification.

- {work_dir}: the directory from which the workflow was called
- {entrypoint_dir}: the directory containing the Icolos entry-point

```
"header": {
    "workflow_id": "Icolos_workflow",
        "description": "Icolos workflow header",
        "environment": {
            "export": [
            {
                "key": "GMX_GPU_DD_COMMS",
                "value": "true"
            },
            {
                "key": "GMX_GPU_PME_PP_COMMS",
                "value": "true"
            },
            {
                "key": "GMX_FORCE_UPDATE_DEFAULT_GPU",
                "value": "true"
            }
        },
        "global_variables": {
            "file_base": "{work_dir}/input_files",
            "output_dir": "{work_dir}/output"
        },
        "global_settings": {
            "single_dir": true,
            "remove_temporary_files": false
        }
    }
}
```

Steps

Steps in the workflow are defined and executed sequentially. In general, each step corresponds to the execution of a single program (there are some exceptions to this, for example internal data manipulation steps). Each step is specified using the following fields (the options shown here are for demonstration purposes and do not constitute a 'normal' Glide docking step configuration, see section 3 for specific details):

```
{
    "step_id": "Glide",
    "type": "glide",
```

```
    "execution": {
        "prefix_execution": "module load schrodinger/2021-2-js-aws",
        "parallelization": {
            "cores": 4,
            "max_length_sublists": 1
        },
        "failure_policy": {
            "n_tries": 3
        }
    },
    "settings": {
        "arguments": {
            "flags": ["-WAIT"],
            "parameters": {
                "-HOST": "localhost"
            }
        },
        "additional": {
            "configuration": {
                "AMIDE_MODE": "trans",
                "EXPANDED_SAMPLING": "True",
                "GRIDFILE": ["{work_dir}/test_grid.zip"],
                "NENHANCED_SAMPLING": "1"
            }
        }
    },
    "input": {
        "compounds": [
            {
                "source": "Ligprep",
                "source_type": "step"
            }
        ],
        "generic": {
            {
                "source": "/path/to/receptor.pdb",
                "extension":"pdb"
            }
        }
    },
    "writeout": [
        {
            "compounds": {
                "category": "conformers"
            },
            "destination": {
                "resource": "{work_dir}/conformers.sdf",
                "type": "file",
                "format": "SDF"
            }
```

```
            }

        ]
    }
```

Each step is assigned a `step_id` which is unique within the workflow. Steps can be referenced by their step_id by subsequent steps in the workflow (e.g. when specifying step input). The `type` field identifies which step is being instantiated (multiple steps can be of the same type as long as their identifiers are unique).This must correspond exactly to the step identifier held internally. See section 3 for specific details for each step.

The step's `execution` block captures the settings for executing the step's corresponding program (for example the glide step calls `$SCHRODINGER/glide`, pdb2gmx calls `gmx pdb2gmx`). Settings controlling parallel execution within the step (see section 4.3) are specified in the `parallelization` block, including the number of cores to run on and the maximum length of any single job queue. The parallelizer's failure policy is also defined here.

The `settings` block controls the specifics of how the step is executed. Flags and parameters passed in `settings.arguments` provide direct control over the arguments passed to the program, and will override the internal defaults where applicable. Note that you should only pass arguments that modify step functionality; arguments specifying input files are handled automatically by Icolos.

The `settings.additional` block contains step-specific flags that control the execution of the Icolos step, as opposed to the underlying program. Typically, this field is used to control more complicated features such as handling a filter file for LigPrep steps. See section 3 for details of the specific settings available for each program.

The `input` block specifies where the input data for the step come from. This is divided into Compound and Generic input, see section 4 for further details on how data is handled within Icolos workflows. In this toy example the Compounds (with attached enumerations) from a preceding LigPrep step are read into the Glide step, and an additional PDB structure is read from file and stored using the step's generic file handling methods.

Finally, the `writeout` block handles writing data (including computed properties) to a file. This is similarly divided into Compound and Generic writeout blocks.

1.1.4 Compound handling

A key type of data handled in Icolos workflows are compounds (small molecules). Internally, a 3-tier data structure is used to represent these molecules on different levels:

1. Compound: The top-level entity that represents an input molecule, e.g. read in from a SMILES file. Often, we are interested in more than one variant of a compound, e.g. when dealing with different protonation states. Thus, a compound can be comprised by any number of Enumerations.

2. Enumeration: Each enumeration represents exactly one state (e.g. with defined stereo-chemistry, protonation states or tautomers). Since molecules are also flexible, we need to be able to store different 3D configurations. Thus, an Enumeration can be comprised by any number of Conformers.

3. Conformer: Different 3D configurations (e.g. an ensemble of solvated structures or docking poses) are stored in the Conformer class and associated with an Enumeration.

This slightly complicated structure results in the following naming scheme for Conformers: ⟨compound_number⟩:⟨enumeration_id⟩:⟨conformer_id⟩.

Example

Consider the following example docking protocol: A user feeds in 2 Compounds as SMILES into a workflow. Stereo-enumeration results in 2 Enumerations for the former and 4 for the latter Compound. For each Enumeration, 3 docking poses and their associated docking scores are generated. This would result in 2*3=6 poses (Conformers) for Compound A and 4*3=12 for compound B.

Supplementary Note 2

Workflow Recipes

Many common workflows are provided as example recipes and are available in the examples folder of the main GitHub repo. These can be used without significant further modification by just replacing the input files. In the following sections, selected workflow recipes will be presented and discussed.

2.1 Docking

Icolos enables high throughput molecular docking. Three docking engines are currently supported: Glide, GOLD and AutoDock Vina. For more details, see the individual steps described in Section 3. A docking workflow consists of two steps:

1. Embedding: Generation of enumerations from a SMILES string and assigning 3D coordinates
2. Docking: Generation of docked poses for each enumeration

2.2 Automated SMILES-to-$\Delta\Delta$G

The vast majority of approaches that automated free energy calculations are closed-source solutions. To our knowledge, no such workflow implementation exists that allows interchangeable use of commercial and open-source components for various steps, facilitates job dispatch, monitoring and error checking on a wide variety of hardware (both local and cloud-based), and provides an end-to-end solution to compute $\Delta\Delta$G values from SMILES representations. The current solution represents a key capability for enabling routine calculation of accurate binding affinities and for the incorporation of this knowledge into other applications, for example de-novo design tasks. This multi-step, highly heterogeneous workflow was designed and implemented using the Icolos workflow management platform [1], and enables construction and evaluation of a full set of perturbations in 2-3 days on a typical in-house cluster.

Execution of the workflow starts with generating a configuration file in `json` format, which completely specifies the workflow, including the steps to be executed, their settings and locations of required files. In practice, one of the example configuration files provided in the Icolos public repository should require minimal changes to evaluate a new system and could thus serve as a starting point.

For brevity, we limit our discussion here to the most complex steps and pertinent details. First, we highlight the pmx_setup step, responsible for preparing the calculations for subsequent steps. Notably, the additional settings block controls the key configuration options, including the forcefield used to parameterise the protein, the water model, and the number of replicas. This step instantiates an internal `PerturbationMap` object which encodes the connectivity and identity of the ligands in the perturbation map, with the map log file coming from an earlier LOMAP or fep_setup step (see Section 3.3.1), and compounds from a

previous docking step (see full configuration files provided in the SI for reference). The execution block sets up the environment for executing parameterisation, which involves running `gmx pdb2gmx` on the protein and `ACPYPE` on the ligands. Ligand parametrisation can be run concurrently, by controlling the number of jobs in the `parallelisation` block. Note that any step's jobs can be dispatched to SLURM by applying an execution configuration similar to the transition step provided below.

The required files are provided in the `input` block, including the log file from the fep_mapper step, PDB file for the receptor, and a directory containing GROMACS simulation parameter `.mdp` files for each simulation stage. Compounds, including docked conformers are taken from the preceding docking stage, and a work_dir is specified for storing output for the workflow.

```
{
    "step_id": "01_pmx_setup",
    "type": "pmx_setup",
    "execution": {
        "prefix_execution": "ml GROMACS",
        "parallelization": {
            "jobs": 24
        }
    },
    "settings": {
        "arguments": {
            "flags": [],
            "parameters": {}
        },
        "additional": {
            "water": "tip3p",
            "forcefield":
                "amber99sb-star-ildn-mut",
            "replicas": 3
        }
    },
    "input": {
        "generic": [
            {
                "source": "fep_setup",
                "extension": "log"
            },
            {
                "source": "{file_path}/4ui5.pdb",
                "extension": "pdb"
            },
            {
                "source": "{file_path}/mdp_dir/",
                "extension": "mdp"
            }
        ],
        "compounds": [
            {
                "source": "Glide",
                "source_type": "step"
            }
        ],
        "work_dir": "{output_dir}"
    }
}
```


Below is an example configuration block for a transition simulation step. The execution block controls dispatch and parallelisation of the jobs. By specifying SLURM as the platform, jobs are written to a batch script with the specified resources requested, submitted to a cluster and monitored. In this example case, 128 jobs from the pool will be run through concurrently, with a new job submitted each time one finishes,

until the pool is exhausted.

Flags and parameters from settings are appended to each job command, allowing for a direct control over the underlying program, in this case `gmx mdrun`. Here, we explicitly specify the number of MPI ranks and OMP threads on which to run the job to maximise performance on the allocated 16 cores.

```
{
    "step_id": "09_run_transitions",
    "type": "pmx_run_simulations",
    "execution": {
        "platform": "slurm",
        "resources": {
            "partition": "core",
            "cores": "16",
            "mem": "8g",
            "time": "48:00:00",
            "modules": [
                "GROMACS"
            ]
        },
        "parallelization": {
            "jobs": 128
        }
    },
    "settings": {
        "arguments": {
            "flags": [],
            "parameters": {
                "-ntmpi": "1",
                "-ntomp": "16"
            }
        },
        "additional": {
            "sim_type": "transitions"
        }
    }
}
```

Supplementary Note 3

Workflow Steps

The following sections provide details on the implementation and available options for each step implemented in Icolos.

3.1 Input / Output

3.1.1 Data Manipulation

The data manipulation step provides core functionality to filter, combine or transform workflow data at a specific point in the workflow.
The following actions are supported, and can be selected using the `action` keyword in `settings.additional`:

- `convert_mae_to_pdb`: Useful when constructing a workflow that combines Schrödinger components with, for example, GROMACS.

- `assemble_complexes`: concatenates conformers passed as step input (e.g. from a previous docking step) with a pdb structure. Requires the following `additional` keywords to be set:

  `receptor`: path to pdb file containing receptor (no ligand)

- `filter`: Filter compounds at either conformer or enumeration level based on the criteria provided property. Requires the following `additional` keywords to be set:

  `return_n`: number of results to return at the set filter level (enumeration or conformer)

  `highest_is_best`: bool

  `criteria`: string (or list of strings) matching the property on which to rank and filter

  `aggregation`: aggregation mode if combining multiple criteria. product—sum.

  `filter_level`: level at which to filter: compound|enumeration. If compound, extract at most `return_n` conformers, regardless of enumeration. If enumeration, extract at most `return_n` conformers for each enumeration.

3.2 Molecular Docking

3.2.1 RDKit Embedding

RDKit open-source backend [2] can be used to embed compounds. This provides fewer options than LigPrep [3] to control tautomer and stereo-chemistry. An example configuration is provided below, which takes as input SMILES directly from a text file.

```
{
    "step_id": "rdkit_embedding",
    "type": "embedding",
    "settings": {
        "arguments": {
            "flags": [],
            "parameters": {
                "protonate": true,
                "method": "rdkit"
            }
        },
        "additional": {}
    },
    "input": {
        "compounds": [{
                "source": "/path/to/compounds.smi",
                "source_type": "file",
                "format": "SMI"
            }
        ]
    }
},
```

3.2.2 LigPrep

`LigPrep` allows ligands to be prepared from SMILES strings, generating enumerations over tautomers, protonation states and stereochemistry [3–5]. SMILES can be loaded from a csv file (`header` must be provided to identify the column header containing the SMILES strings), from a string separated by ';' or from a `.smi` file.
A sample configuration is provided below:

```
{
     "step_id": "Ligprep",
     "type": "ligprep",
     "execution": {
          "prefix_execution": "module load schrodinger/2021-4",
          "parallelization": {
               "cores": 3,
               "max_length_sublists": 2
          },
          "failure_policy": {
               "n_tries": 3
          }
     },
     "settings": {
          "arguments": {
               "flags": ["-epik"],
               "parameters": {
                    "-ph": 7.0,
                    "-pht": 2.0,
                    "-s": 10,
```

```
                    "-bff": 14
                }
            },
            "additional": {
            }
        },
        "input": {
            "compounds": [{
                    "source": "initialization_smile",
                    "source_type": "step"
                }
            ]
        }
}
```

The following additional keywords are available

- `filter_file`: dictionary, allows specification of the filter file

3.2.3 AutoDock Vina

`AutoDock Vina` is a widely used, freely available docking backend. There are several flavours available, but in `Icolos` we integrate `AutoDock Vina 1.2.0` which can be obtained here: ADV github. While there is a GPU version available as well, we have opted to use the latest CPU-based iteration, which is fully parallelized [6, 7].

AutoDock Vina target preparation

In order to use `AutoDock Vina`, one needs to prepare a receptor structure first. A reference ligand is used to obtain the location and dimensions of the pocket, thus serving as guidance for the molecular docking. Before the target is constructed, make sure that the structure is well prepared (e.g. take care of missing side-chains etc.). `AutoDock Vina` requires the target to be prepared in the `PDBQT` format, which is very similar to the `PDB` format but contains `Gasteiger charges` as well. You can generate your receptor with the `vina_target_preparation` step in `Icolos`:

```
{
    {
        "step_id": "ADV_target_prep",
        "type": "vina_target_preparation",
        "execution": {
        },
        "settings": {
            "arguments": {
                "flags": [],
                "parameters": {
                }
            },
            "additional": {
                "pH": 7.4,
            "input_receptor_pdb": "/path/to/input_structure.pdb",
            "output_receptor_pdbqt": "/path/to/output_structure.pdbqt",
```

```
            "extract_box": {
                "reference_ligand_path": "/path/to/reference_ligand.pdb",
                "reference_ligand_format": "PDB"
            }
            }
        }
    }
}
```

This uses the following additional parameters:

- `pH`: Affects the charge state.
- `input_receptor_pdb`: Path to the prepared input `PDB` file - without the reference ligand!
- `output_receptor_pdbqt`: Output path the final `PDBQT` file will be written to.
- `extract_box`: Defines the specification of the box (i.e. the binding pocket) based on a reference ligand.
  - `reference_ligand_path`: Path to the file that holds the reference ligand's coordinates.
  - `reference_ligand_format`: Either `PDB` or `SDF` file format.

Note, that this step logs the centroid location and minimum and maximum values in XYZ on an `INFO` level to assist you with finding appropriate settings for docking (see 3.2.3). This looks like the following:

```
2021-12-14 15:20:17 - Ligand (/path/to/reference_ligand.pdb):
X coordinates: min=-2.44, max=8.12, mean=3.19
Y coordinates: min=7.27, max=15.53, mean=11.48
Z coordinates: min=23.0, max=26.95, mean=24.76
```

AutoDock Vina docking

Docking is a two-step process (embedding of ligands in 3D, molecular docking) so the following assumes that the prospective ligands have already been embedded, for example using the `Embedder` (see 3.2.1 for details) or `Ligprep`.

```
{
    "step_id": "ADV",
    "type": "vina_docking",
    "execution": {
        "binary_location": "/path/to/vina/folder",
        "parallelization": {
            "cores": 4
        },
        "failure_policy": {
            "n_tries": 3
        }
    },
    "settings": {
        "arguments": {
            "flags": [],
```

```
            "parameters": {
            }
        },
        "additional": {
            "configuration": {
                "seed": 42,
                "receptor_path": "/path/to/receptor.pdbqt",
                "number_poses": 2,
                "search_space": {
                    "--center_x": 3.2,
                    "--center_y": 11.5,
                    "--center_z": 24.8,
                    "--size_x": 15,
                    "--size_y": 10,
                    "--size_z": 10
                }
            },
            "grid_ids": ["1UYD"]
        }
    },
    "input": {
        "compounds": [{
                "source": "Ligprep",
                "source_type": "step"
            }
        ]
    },
    "writeout": [
        {
            "compounds": {
                "category": "conformers"
            },
            "destination": {
                "resource": "/output/path/adv_docked_conformers.sdf",
                "type": "file",
                "format": "SDF"
            }
        }
    ]
}
```

This uses the following additional parameters:

- `configuration`:
  - `seed`: Random seed, ensures reproducability if set to the same number.
  - `receptor_path`: Path to the `PDBQT` file holding the receptor (see 3.2.3 for details).
  - `number_poses`: Integer value > 0 that controls how many poses are reported for each `Enumeration`.
  - `search_space`: Defines which region of the target protein is of interest for docking (see 3.2.3 for instructions on how to select the values based on a reference ligand).

* `--center_x`: Center of search space, X-coordinate (float).
* `--center_y`: Center of search space, Y-coordinate (float).
* `--center_z`: Center of search space, Z-coordinate (float).
* `--size_x`: Length of search "box" in X-direction (float).
* `--size_y`: Length of search "box" in Y-direction (float).
* `--size_z`: Length of search "box" in Z-direction (float).

3.2.4 Glide

`Glide` is one of the most popular molecular docking backends, showing excellent performance on virtual screening tasks. Icolos implements fully parallelized support for Glide [8–11].

Protein preparation

A docking run requires a grid file to be prepared for the protein beforehand. This is most commonly done using the `Maestro` GUI, and captures the topology of the binding site and allows for receptor-based constraints to be specified.

Docking configuration

A docking configuration file can either be provided explicitly in `settings.additional.maestro_in_file`, or provided as a list of `key:value` pairs as they would appear in a `.in` file produced by `Maestro`.
Glide requires a list of compounds with enumerations over tautomers, protonation states, stereochemistry etc. attached, normally using a preceeding `Embedder` or `LigPrep` step (see Sections 3.2.1, 3.2.2).
A Glide docking step looks like the following:

```
{
    "step_id": "Glide",
    "type": "glide",
    "execution": {
        "prefix_execution": "module load schrodinger",
        "parallelization": {
            "cores": 4,
            "max_length_sublists": 1
        },
        "failure_policy": {
            "n_tries": 3
        }
    },
    "settings": {
        "arguments": {
            "flags": [],
            "parameters": {
                -HOST: "localhost"
            }
        },
        "additional": {
            "configuration": {
                "AMIDE_MODE": "trans",
                "EXPANDED_SAMPLING": "True",
```

```
                "GRIDFILE": [
                    "/path/to/grid.zip"
                ],
                "NENHANCED_SAMPLING": "1",
                "POSE_OUTTYPE": "ligandlib_sd",
                "POSES_PER_LIG": "3",
                "POSTDOCK_NPOSE": "25",
                "POSTDOCKSTRAIN": "True",
                "PRECISION": "SP",
                "REWARD_INTRA_HBONDS": "True"
            }
        }
    },
    "input": {
        "compounds": [
            {
                "source": "Ligprep",
                "source_type": "step"
            }
        ]
    },
    "writeout": [
        {
            "compounds": {
                "category": "conformers",
                "selected_tags": [
                    "docking_score",
                    "grid_id"
                ],
                "aggregation": {
                    "mode": "best_per_compound",
                    "key": "docking_score",
                    "highest_is_best": false
                }
            },
            "destination": {
                "resource": "/path/to/conformers.csv",
                "type": "file",
                "format": "CSV"
            }
        }
    ]
}
```

This step accepts the following additional settings:

- `maestro_in_file`: Accepts a maestro file instead of parsing from the configuration dictionary
- `time_limit_per_task`: Maximum time to allow per compound before aborting the job. Default 120 seconds
- `configuration`: Alternative to `maestro_in_file`, Glide configuration specified by `key:value` pairs.

- `grid_ids`: identifiers for the grids if using ensemble docking against multiple receptor grids.

Ensemble Docking

Icolos supports ensemble docking, where a set of ligands is docked against multiple receptor grids. Commonly these are constructed from different crystal structures, and capture some of the flexibility of the receptor's binding pocket. In order to perform ensemble docking, a list of grid paths should be passed as the `GRIDFILE` value in `settings.additional.config`. Identifiers for these grids can be passed using the `grid_ids` setting.
Note that ensemble docking is often executed with a subsequent data manipulation step to automatically filter down to the best docking score per compound across multiple grids. See 3.1.1 for details.

3.3 Free Energy Methods

3.3.1 Perturbation Map Generation

Icolos provides two backends to generate perturbation maps: Schrödinger's `fep_mapper.py` [12, 13] script and `LOMAP` [14]. The topology generated by both is stored using a common PerturbationMap class within the workflow, such that either step can be substituted into the workflow without further modification. Example step configurations for both options are shown below, including the required input files in each case.

For `LOMAP`, conformers are extracted directly from a previous docking step (using Glide in the example below), and we specify the topology as either `mcs` (the default) or `radial`.

```
{
    "step_id": "step_lomap_perturbation_mapper",
    "type": "lomap",
    "execution": {
        "parallelization": {
            "jobs": 24
        }
    },
    "settings": {
        "arguments": {
            "flags": [],
            "parameters": {}
        },
        "additional": {
            "topology": "mcs"
        }
    },
    "input": {
        "compounds": [
            {
                "source": "Glide",
                "source_type": "step"
            }
        ]
    },
}
```

Here we show an example `fep_mapper` step used to generate a radial map with a specified hub node. Note the use of arguments and parameters to directly control the options passed to the program. Here we provide a separate reference ligand as an sdf file, as well as conformers taken from a previous Glide step, and the pdb file containing the receptor. Both of these configurations can be used interchangeably in a free energy workflow.

```
{
    "step_id": "fep_setup",
    "type": "fep_plus_setup",
    "execution": {
        "prefix_execution": "module load schrodinger/2021-4-js-aws"
    },
    "settings": {
        "arguments": {
            "parameters": {
                "-num-procs": 1,
                "-t": "star",
                "-add-bias": "ref_lig",
                "-bias": "ref_lig"
            }
        }
    },
    "input": {
        "compounds": [
            {
                "source": "{file_path}/ref_lig.sdf",
                "source_type": "file"
            },
            {
                "source": "Glide",
                "source_type": "step"
            }
        ],
        "generic": [
            {
                "source": "{file_path}/example.pdb",
                "extension": "pdb"
            }
        ]
    }
},
```

3.3.2 PMX

Icolos supports automated computation of relative binding free energies (RBFE) using the open-source PMX package [15, 16], and the GROMACS MD engine [17, 18]. To facilitate this, we created multiple entrypoints in PMX compatible with Icolos data structures and parallelization schemes. Both the Icolos wrappers and the PMX implementations are discussed here.

In general, a PMX RBFE workflow consists of the following steps executed sequentially.

- PMX setup
- atomMapping
- ligandHybrid
- assembleSystems
- boxWaterIons
- prepareSimulations
- runSimulations
- prepareTransitions
- runTransitions
- analyse

PerturbationMap

Perturbation maps can be generated and analysed using either Schrödinger's `fep_mapper` script [13] or LOMAP [14]. The resulting map's connectivity is parsed from the respective program's log file to an internal `PerturbationMap` object. This object is central to the subsequent PMX workflow, and is used by all steps to access the map topology.

The `PerturbationMap` holds a list of nodes and edges describing the connectivity of the map, and tracks node identities using unique hashes.

PMXSetup

PMX workflows are uniquely executed in a single directory, since it is impractical to store large amounts of simulation data in memory using Icolos' standard file management. Instead, a work_dir is established in the `PMXSetup`, with each subsequent step operating on the same data.

`PMXSetup` is responsible for setting up a fresh work_dir, taking as input the compounds and perturbation map log file from a previous FEPSetup step, in addition to a receptor structure. `PMXSetup` generates Amber forcefield parameters for the compounds and receptor (using Antechamber and pdb2gmx calls, respectively) and creates the folder structure inside the `work_dir`.

Additionally, the number of replicas to be used throughout the workflow is set in this step, using `settings.additional.replicas`. If unset, the default of 3 is used.

```
{
    "step_id": "01_pmx_setup",
    "type": "pmx_setup",
    "execution": {
        "prefix_execution": "module load GROMACS",

        "parallelization": {
            "jobs": 24
        }
    },
```

```
    "settings": {
        "arguments": {
            "flags": [],
            "parameters": {}
        },
        "additional": {
            "water": "tip3p",
            "forcefield": "amber99sb-star-ildn-mut",
            "replicas": 3
        }
    },
    "input": {
        "generic": [
            {
                "source": "fep_setup",
                "extension": "log"
            },
            {
                "source": "{file_path}/4ui5.pdb",
                "extension": "pdb"
            },
            {
                "source": "{file_path}/pmx/run_simulations/input/mdp/",
                "extension": "mdp"
            }
        ],
        "compounds": [
            {
                "source": "fep_setup",
                "source_type": "step"
            }
        ],
        "work_dir": "{output_dir}"
    }
},
```

PMXatomMapping

This step directly calls the atomMapper program from the PMX installation, which identifies pairs of atoms between the end states that require morphing via alchemical transformation, and returns PDB files containing the appropriate dummy atoms.

```
{
    "step_id": "02_pmx_atomMapping",
    "type": "pmx_atomMapping",
    "execution": {
        "parallelization": {
            "jobs": 24
        }
    },
    "settings": {
```

```
        "arguments": {
            "flags": [],
            "parameters": {}
        },
        "additional": {
            "water": "tip3p",
            "forcefield": "amber99sb-star-ildn-mut"
        }
    }
},
```

PMXligandHybrid

A direct call to the pmx ligandHybrid program, builds hybrid structures and topologies for the two end states

```
{
    "step_id": "03_pmx_ligandHybrid",
    "type": "pmx_ligandHybrid",
    "execution": {
        "prefix_execution": "module load GROMACS/2021-fosscuda-2019a-PLUMED-2.7.1-Python-3.7.2"
    },
    "settings": {
        "arguments": {
            "flags": [],
            "parameters": {
                "-cs": "spc216.gro"
            }
        },
        "additional": {}
    }
},
```

PMXassembleSystems

Calls the `assemble_systems.py` entry-point from PMX, generates a merged topology for the protein-ligand complex including the dummy atoms introduced in previous steps

```
{
    "step_id": "04_assemble_systems",
    "type": "pmx_assemble_systems",
    "execution": {
        "prefix_execution": "module load GROMACS/2021-fosscuda-2019a-PLUMED-2.7.1-Python-3.7.2",
        "binary_location": "\$PMX_PYTHON <fill_pmx_path>/src/pmx/scripts/icolos_entrypoints/",
        "parallelization": {
            "jobs": 24
        }
    },
    "settings": {
        "arguments": {
            "flags": [],
```

```
            "parameters": {}
        },
        "additional": {
        }
    }
},
```

PMXboxWaterIons

Calls the `box_water_ions.py` entrypoint from PMX. For each structure generated (4 per edge), a water box, solvent and neutralizing salt is added, using successive calls to `gmx editconf, solvate, genion`. Since only basic gromacs functionality is required here, the wrappers included with PMX are preferred to Icolos'.

```
{
    "step_id": "05_box_water_ions",
    "type": "pmx_box_water_ions",
    "execution": {
        "prefix_execution": "module load GROMACS/2021-fosscuda-2019a-PLUMED-2.7.1-Python-3.7.2",
        "parallelization": {
            "jobs": 24
        }
    },
    "settings": {
        "arguments": {
            "flags": [],
            "parameters": {}
        },
        "additional": {}
    }
},
```

PMXprepareSimulations

Calls the `prepare_simulations.py` entrypoint from PMX. Produces a run time `.tpr` file for each simulation through a `gmx grompp` call. This step is called for both the equilibration and production simulations.

```
{
    "step_id": "06_prepare_simulations",
    "type": "pmx_prepare_simulations",
    "execution": {
        "prefix_execution": "module load GROMACS/2021-fosscuda-2019a-PLUMED-2.7.1-Python-3.7.2",
        "parallelization": {
            "jobs": 24
        }
    },
    "settings": {
        "arguments": {
            "flags": [],
            "parameters": {}
        },
```

```
        "additional": {
            "sim_type": "em"
        }
    }
},
```

PMXrunSimulation

Runs the simulations, either using local resources allocated to the Icolos job (if `platform` = `local`), or, most commonly, distributing the jobs to SLURM. In the latter case job scripts are generated using the specified resources which are submitted and monitored. In the example below the parallelisation scheme ensures a maximum of 96 jobs are running simultaneously, until the job pool is exhausted. Flags and parameters can be used to provide additional control over the underlying `gmx mdrun` call, in this case to specify threading options.

```
{
    "step_id": "06d_run_simulations",
    "type": "pmx_run_simulations",
    "execution": {
        "platform": "slurm",
        "resources": {
            "partition": "core",
            "cores": 16,
            "mem": "8g",
            "modules": [
                "GROMACS/2021-fosscuda-2019a-PLUMED-2.7.1-Python-3.7.2"
            ]
        },
        "parallelization": {
            "jobs": 96
        }
    },
    "settings": {
        "arguments": {
            "flags": [],
            "parameters": {
                "-ntomp": 16,
                "-ntmpi": 1
            }
        },
        "additional": {
            "sim_type": "nvt"
        }
    }
},
```

PMXprepareTransitions

Handles frame extraction from the equilibrium simulations, subsequently prepares tpr files for each transition to be executed (actual simulation is still handled by `pmx_run_simulations` by specifying `sim_type` = `transitions`. In the step configuration below, flags and parameters, for example `-b`, are passed directly

to the `gmx trjconv` call for frame extraction.

As with all pmx-related steps, transition preparation is parallelised over the edges of the perturbation map.

```
{
    "step_id": "08_prep_transitions",
    "type": "pmx_prepare_transitions",
    "execution": {
        "prefix_execution": "module load GROMACS/2021-fosscuda-2019a-PLUMED-2.7.1-Python-3.7.2",
        "parallelization": {
            "jobs": 24
        }
    },
    "settings": {
        "arguments": {
            "flags": [],
            "parameters": {
                "-b": 2000
            }
        },
        "additional": {
        }
    }
},
```

PMXrunAnalysis

The final step analyses the TI data produced from the transitions, generates summary csv files and attaches results to the internal Compound list. This step calls the `pmx analyse` program under the hood. An example configuration is provided below.

```
{
    "step_id": "pmx_analyse",
    "type": "pmx_run_analysis",
    "execution": {
        "parallelization": {
            "jobs": 24
        }
    },
    "settings": {
        "arguments": {
            "flags": [],
            "parameters": {}
        },
        "additional": {}
    }
}
```

Supplementary Note 4

Implementation Details

4.1 Data Handling

Icolos handles all data passed to and generated during a workflow using two separate mechanisms: Compounds and Generic.

4.1.1 Compound Objects

Compounds are tracked through the workflow and passed from step to step, with computed properties added as tags as the workflow progresses. See also the description in 1.1.4. The highest level container is Compound object, corresponding to a single SMILES string. For each enumeration, specific poses are stored as Conformer objects. This class wraps an RDkit Mol object to store the coordinates. Computed conformer properties are attached as tags throughout the workflow.

In general, Compounds (and by implication their attached Enumerations and Conformers) are passed through the workflow from one step to the next.

4.1.2 GenericData

Some steps (and sometimes entire workflows) do not operate on Compound-type objects only, and in this case the GenericContainer class is used to store, index and retrieve files (stored as GenericData objects) required for the execution of each step.

Plain text or binary files can be read from the file system, from a previous step, or from an API using a similar syntax to Compounds in the configuration file (**Section 1.1.3**).

4.2 Execution

Icolos executes the underlying program, either through Python's `subprocess` module, or using a SLURM interface to execute and monitor a batch job. Execution settings are controlled through the step's `execution` block. An example is provided below (some blocks replaced by "..." for brevity):

```
{
    "execution": {
        "binary_location": ...
        "prefix_execution": ...
        "resource": "slurm",
        "job_control": {
```

```
            "partition": "gpu",
            "gres": "gpu:1",
            "modules": [
                "GROMACS/2021-fosscuda-2019a-PLUMED-2.7.1-Python-3.7.2"
            ]
        }
    }
}
```

binary_location is used to point the directory containing the binary, which avoids having to add all the programs to be used to $PATH prior to executing the workflow. Similarly, for programs that require a module to be loaded, or some other command to be performed prior to the call (e.g. sourcing a patricular file), prefix_execution can be used. Together, these options allow for creating an isolated execution environment and reduce issues with conflicting software loaded into the environment.
If a resource block is not specified, the job defaults to running on the same resources the workflow itself is running on. If execution.resource='slurm' is set, a batch script will be created using the parameters specified in execution.job_control, which is then executed and monitored by the Icolos process. This allows for compute-intensive processes to be run in parallel using their own isolated resources, thus facilitating execution and scaling of free energy calculations.

4.3 Parallelization

Icolos uses Python's multiprocessing module to perform parallel execution of steps that operate on many compounds/enumerations/conformers and are therefore trivially parallelizable. In a typical docking workflow, a batch of compounds are enumerated from SMILES strings to embedded molecules, enumerating over tautomers, stereoisomers, probable charge states etc. Each enumeration is then docked into the receptor grid using one of the supported docking engines (Glide, ADV, Gold) to generate a set of conformers per enumeration.

During enumeration, compounds are divided across the number of specified cores and run in parallel. Similarly for the docking step, enumerations are unrolled and distributed across multiple cores, and the compounds are reconstructed afterwards, now with attached conformers and docking scores.

All steps that operate on compounds are parallelized in the same way, so specifying n cores in the step's execution.parallelization block will provide an (almost) n-fold speedup compared to single-core performance.

In the configuration shown below, taken from a Glide docking step, enumerations are divided into parallel queues over 4 cores, each queue containing a maximum of 12 separate enumerations to be docked (which can be helpful for very large input data). Each set of 48 enumerations is processed as a single batch, and repeated until the queue has been exhausted.

A failure policy is also specified, which allows failed jobs to be resubmitted several times in case of a runtime issue (this is particularly useful when submitting jobs to run on remote hosts where random failures of this kind are to be expected).

```
{
    "parallelization": {
        "cores": 4,
        "max_length_sublists": 12
```

```
    },
    "failure_policy": {
        "n_tries": 3
    }
}
```

Supplementary references

1. Moore, J. H. *et al.* Icolos: A workflow manager for structure based post-processing of de novo generated small molecules. en. *Chemrxiv.* https://chemrxiv.org/engage/chemrxiv/article-details/61f3c957537af8ea2ea80fba (2022) (Feb. 2022).
2. Landrum, G. *RDKit: Open-source cheminformatics; http://www.rdkit.org* Apr. 2022. https://zenodo.org/record/6483170 (2022).
3. *Schrödinger Release 2022-1: LigPrep, Schrödinger, LLC, New York, NY, 2021.*
4. Shelley, J. C. *et al.* Epik: a software program for pK a prediction and protonation state generation for drug-like molecules. en. *Journal of Computer-Aided Molecular Design* **21,** 681–691. ISSN: 0920-654X, 1573-4951. http://link.springer.com/10.1007/s10822-007-9133-z (2022) (Dec. 2007).
5. Greenwood, J. R., Calkins, D., Sullivan, A. P. & Shelley, J. C. Towards the comprehensive, rapid, and accurate prediction of the favorable tautomeric states of drug-like molecules in aqueous solution. en. *Journal of Computer-Aided Molecular Design* **24,** 591–604. ISSN: 0920-654X, 1573-4951. http://link.springer.com/10.1007/s10822-010-9349-1 (2022) (June 2010).
6. Eberhardt, J., Santos-Martins, D., Tillack, A. F. & Forli, S. AutoDock Vina 1.2.0: New Docking Methods, Expanded Force Field, and Python Bindings. *Journal of Chemical Information and Modeling* **61.** Publisher: American Chemical Society, 3891–3898. ISSN: 15205142. https://pubs.acs.org/doi/full/10.1021/acs.jcim.1c00203 (2022) (Aug. 2021).
7. Trott, O. & Olson, A. J. AutoDock Vina: Improving the speed and accuracy of docking with a new scoring function, efficient optimization, and multithreading. en. *Journal of Computational Chemistry* **31.** _eprint: https://onlinelibrary.wiley.com/doi/pdf/10.1002/jcc.21334, 455–461. ISSN: 1096-987X. https://onlinelibrary.wiley.com/doi/abs/10.1002/jcc.21334 (2022) (2010).
8. *Schrödinger Release 2022-1: Glide, Schrödinger, LLC, New York, NY, 2021.*
9. Friesner, R. A. *et al.* Extra Precision Glide: Docking and Scoring Incorporating a Model of Hydrophobic Enclosure for ProteinLigand Complexes. en. *Journal of Medicinal Chemistry* **49,** 6177–6196. ISSN: 0022-2623, 1520-4804. https://pubs.acs.org/doi/10.1021/jm051256o (2022) (Oct. 2006).
10. Halgren, T. A. *et al.* Glide: A New Approach for Rapid, Accurate Docking and Scoring. 2. Enrichment Factors in Database Screening. en. *Journal of Medicinal Chemistry* **47,** 1750–1759. ISSN: 0022-2623, 1520-4804. https://pubs.acs.org/doi/10.1021/jm030644s (2022) (Mar. 2004).
11. Friesner, R. A. *et al.* Glide: A New Approach for Rapid, Accurate Docking and Scoring. 1. Method and Assessment of Docking Accuracy. en. *Journal of Medicinal Chemistry* **47,** 1739–1749. ISSN: 0022-2623, 1520-4804. https://pubs.acs.org/doi/10.1021/jm0306430 (2022) (Mar. 2004).
12. Fratev, F. & Sirimulla, S. An Improved Free Energy Perturbation FEP+ Sampling Protocol for Flexible Ligand-Binding Domains. en. *Scientific Reports* **9,** 16829. ISSN: 2045-2322. http://www.nature.com/articles/s41598-019-53133-1 (2022) (Dec. 2019).
13. Wang, L. *et al.* Accurate and Reliable Prediction of Relative Ligand Binding Potency in Prospective Drug Discovery by Way of a Modern Free-Energy Calculation Protocol and Force Field. *Journal of the American Chemical Society* **137,** 2695–2703. ISSN: 0002-7863, 1520-5126 (Feb. 2015).

14. Liu, S. *et al.* Lead Optimization Mapper: Automating Free Energy Calculations for Lead Optimization. *Journal of computer-aided molecular design* **27,** 10.1007/s10822-013-9678–y. ISSN: 0920-654X (Sept. 2013).

15. Gapsys, V., Michielssens, S., Seeliger, D. & de Groot, B. L. pmx: Automated protein structure and topology generation for alchemical perturbations. en. *Journal of Computational Chemistry* **36.** _eprint: https://onlinelibrary.wiley.com/doi/pdf/10.1002/jcc.23804, 348–354. ISSN: 1096-987X. `https://onlinelibrary.wiley.com/doi/abs/10.1002/jcc.23804` (2022) (2015).

16. Gapsys, V. *et al.* Large scale relative protein ligand binding affinities using non-equilibrium alchemy. en. *Chemical Science* **11,** 1140–1152. ISSN: 2041-6520, 2041-6539. `http://xlink.rsc.org/?DOI=C9SC03754C` (2022) (2020).

17. Abraham, M. J. *et al.* Gromacs: High performance molecular simulations through multi-level parallelism from laptops to supercomputers. *SoftwareX* **1-2.** Publisher: Elsevier B.V., 19–25. ISSN: 23527110 (2015).

18. Kutzner, C. *et al.* GROMACS in the Cloud: A Global Supercomputer to Speed Up Alchemical Drug Design. *Journal of Chemical Information and Modeling* **62,** 1691–1711. ISSN: 1549-9596, 1549-960X (Apr. 2022).
